# Supplementary material for: Transcriptomic analysis of melon/squash graft junction reveals molecular mechanisms potentially underlying the graft union development
Source: PeerJ. 2021 Dec 13;9:e12569. doi: 10.7717/peerj.12569 (PMC8675255; doi:10.7717/peerj.12569)
Supplement: Supplemental Information 1 [file peerj-09-12569-s001.docx]

Figure S1 The lignin content of graft junction tissue at the IL, CA and VB stage underlying the graft union development. Different letters over the bars denote significance at P < 0.05 by ANOVA.

Table S1 Summary of transcriptome sequencing data generated from nine cDNA libraries.

Table S2 The classification of the enriched GO.

Table S3 The information of KEGGs enrichment.

Table S4 The expression level of plant hormone signaling transduction pathway-related genes.

Table S5 The expression level of lignin biosynthesis pathway-related genes.

Table S6 The specific primers used in fluorescence quantitative PCR detection.
