# Supplementary figures and images for: Transcriptomic analysis of melon/squash graft junction reveals molecular mechanisms potentially underlying the graft union development
Source: PeerJ. 2021 Dec 13;9:e12569. doi: 10.7717/peerj.12569 (PMC8675255; doi:10.7717/peerj.12569)

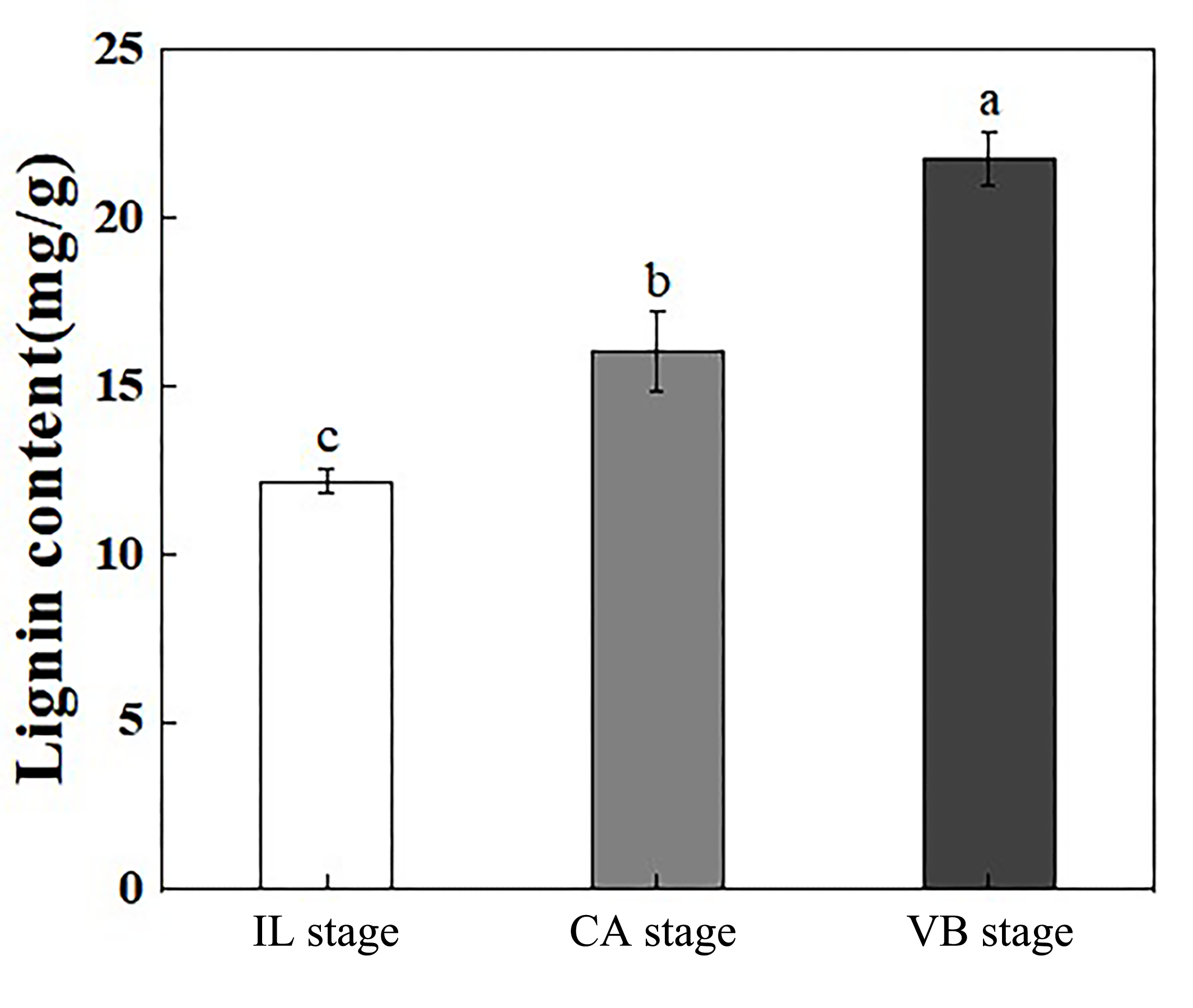

Supplement: Supplemental Information 2 [file peerj-09-12569-s002.png]
