# Supplementary material for: Transcriptomic analysis of melon/squash graft junction reveals molecular mechanisms potentially underlying the graft union development
Source: PeerJ. 2021 Dec 13;9:e12569. doi: 10.7717/peerj.12569 (PMC8675255; doi:10.7717/peerj.12569)
Supplement: Supplemental Information 3 [file peerj-09-12569-s003.docx]

**Table S1** Summary of transcriptome sequencing data generated from nine cDNA libraries.

| Sample Name | Total Reads | Clean Reads | Mapped Reads | | Uniq Mapped Reads | | %≥Q30 |
| --- | --- | --- | --- | --- | --- | --- | --- |
|  |  |  | scion | stock | scion | stock |  |
| IL-1 | 39,732,480 | 19,866,240 | 4,016,303 (10.11%) | 30,102,481 (75.76%) | 3,922,733 (9.87%) | 29,276,243 (73.68%) | 94.05% |
| IL-2 | 47,994,798 | 23,997,399 | 4,591,690 (9.57%) | 36,869,472 (76.82%) | 4,482,099 (9.34%) | 35,906,705 (74.81%) | 94.11% |
| IL-3 | 45,821,434 | 22,910,717 | 5,067,072 (11.06%) | 34,336,578 (74.94%) | 4,963,343 (10.83%) | 33,454,608 (73.01%) | 93.76% |
| CA-1 | 46,419,326 | 23,209,663 | 9,781,579 (21.07%) | 31,192,759 (67.20%) | 9,626,859 (20.74%) | 30,439,061 (65.57%) | 93.80% |
| CA-2 | 45,525,696 | 22,762,848 | 9,415,840 (20.68%) | 30,913,133 (67.90%) | 9,223,807 (20.26%) | 30,075,330 (66.06%) | 94.01% |
| CA-3 | 50,079,018 | 25,039,509 | 11,234,909 (22.43%) | 33,194,634 (66.28%) | 11,025,567 (22.02%) | 32,334,196 (64.57%) | 93.74% |
| VB-1 | 44,741,948 | 22,370,974 | 12,484,552 (27.90%) | 26,927,989 (60.19%) | 12,312,963 (27.52%) | 26,305,564 (58.79%) | 93.66% |
| VB-2 | 43,456,110 | 21,728,055 | 10,644,785 (24.50%) | 27,725,819 (63.80%) | 10,484,852 (24.13%) | 27,076,818 (62.31%) | 93.46% |
| VB-3 | 58,956,572 | 29,478,286 | 14,624,101 (24.80%) | 37,366,148 (63.38%) | 14,408,080 (24.44%) | 36,484,919 (61.88%) | 93.84% |
