# Supplementary material for: Transcriptomic analysis of melon/squash graft junction reveals molecular mechanisms potentially underlying the graft union development
Source: PeerJ. 2021 Dec 13;9:e12569. doi: 10.7717/peerj.12569 (PMC8675255; doi:10.7717/peerj.12569)
Supplement: Supplemental Information 8 [file peerj-09-12569-s008.docx]

**Table S5** The list of specific primers used in fluorescence quantitative PCR detection

| （Gene） | （Primer sequences） |
| --- | --- |
| *MELO3C015880.2* | (F)GGGAGATGGCTGGGAGTAATGA  (R)ACGGCGGAGGATGGTGTAA |
| *MELO3C004382.2* | (F)CGCAACAACCTAACGATT  (R)GACGACGACACTCCTAAG |
| *MELO3C007691.2* | (F)TGACGACTCCTCTACCAA  (R) TACGCATCTGAACCACAA |
| *CmoCh07G009530* | (F) GATGTGATGGTCGAGTTGG  (R) GAGCTCCACCTTGATTGG |
| *MELO3C014091.2* | (F) AATTCCGGATGATGGAAAGG  (R) CAGCTTTGGTTGCTAGGG |
| *CmoCh08G005650* | (F) CGGCGATGTTTACTCATACC  (R) CATAATAGCACCGCGATACG |
| *CmoCh08G003030* | (F) CAAGACTGCCCTTGTAACG  (R) GTCCACTTTACGCAGATATGG |
| *CmoCh20G005830* | (F) GACAAGAACACCGAGAAGG  (R) CGTGCCCTTTGACTTCC |
| *MELO3C015359.2* | (F) GCAAACAAAGTGCAGATGG  (R) TTGAGGCATTGGCATCC |
| *MELO3C034560.2* | (F) GCTGTTCATCCTCTTGATCC  (R) GTCCTCCAAAGTTGCTTCC |
| *MELO3C010317.2* | (F) TGAAGATGGAGGGAGTGG  (R) TTCGTTCTCAGCAAGTAACC |
| *MELO3C026019.2* | (F) ACCGACTCGCTAACTACC  (R) GACTGAAGGTTGCATCGG |
